# Supplementary material for: Genome-Wide Identification and Analysis of CC-NBS-LRR Family in Response to Downy Mildew and Black Rot in Chinese Cabbage
Source: Int J Mol Sci. 2021 Apr 20;22(8):4266. doi: 10.3390/ijms22084266 (PMC8074028; doi:10.3390/ijms22084266)
Supplement: Supplementary file 1 [file ijms-22-04266-s001.zip › ijms-1145046-supplementary.pdf]

Table S1 The homologous gene of *BrCC-NBS-LRRs* in *Arabidopsis thaliana* and their pusitive function

Gene No.	Homologue Gene in *Ar*.	Function of *Ar*. genes
Bra019755<br>Bra026923<br>Bra026924<br>Bra026977<br>Bra026978	AT1G12210	RFL1 has high sequence similarity to the adjacent disease resistance (R) gene RPS5
Bra002495<br>Bra016781<br>Bra016782<br>Bra016785	AT1G12220	Disease resistance protein (CC-NBS-LRR class) family
Bra019752<br>Bra019754<br>Bra015597	At1g12280	Encodes a NB-LRR protein SUMM2 involved in defense response to bacterium
Bra026979	AT1G12290	Disease resistance protein (CC-NBS-LRR class) family
Bra018834<br>Bra018835<br>Bra018863<br>Bra026094	At1g15890	Disease resistance protein (CC-NBS-LRR class) family
Bra027866	AT1G58410	Disease resistance protein (CC-NBS-LRR class) family
Bra035424<br>Bra029405<br>Bra013134	At1g59620	Disease resistance protein (CC-NBS-LRR class) family
Bra027097	AT1G61180	LRR and NB-ARC domains-containing disease resistance protein;

Bra036995	AT1G61190	Disease resistance protein (CC-NBS-LRR class) family
Bra031482	AT1G63350	NB-ARC domain-containing disease resistance protein
Bra027332	AT3G14470	NB-ARC domain-containing disease resistance protein
Bra037123	AT3G46730	NB-ARC domain-containing disease resistance protein
Bra036845	AT3G50950	Encodes a canonical CC-type NLR protein that is required for the recognition of the T3SE HopZ1a from the pathogenic bacteria *P. syringae*
Bra030778	AT4G10780	LRR and NB-ARC domains-containing disease resistance protein;(source:Araport11)
Bra013947	AT4G26090	Resistant to *P. SYRINGAE 2*, RPS2
Bra026368<br>Bra019063	AT4G27190	NB-ARC domain-containing disease resistance protein
Bra034631	AT4G27220	NB-ARC domain-containing disease resistance protein
Bra011432	AT4G33300	Encodes a member of the ADR1 family nucleotide-binding leucine-rich repeat (NB-LRR) immune receptors
Bra013213	AT5G48620	Disease resistance protein (CC-NBS-LRR class) family
Bra030779<br>Bra017572<br>Bra026682<br>Bra018245	AT5G63020	Disease resistance protein (CC-NBS-LRR class) family
Bra009882<br>Bra037139	AT5G66900	Disease resistance protein (CC-NBS-LRR class) family

Table S2. The Chromosome locations of CC-NBS-LRR in *Arabidopsis thaliana* and Chinese cabbage.

	Chr.1	Chr.2	Chr.3	Chr.4	Chr.5	Chr.6	Chr.7	Chr.8	Chr.9	Chr.10

Arabidopsis thaliana	16	0	0	0	8	_	_	_	_	_
Brassica rapa	5	1	4	0	2	8	0	6	12	2

Table S3. *CC-NBS-LRR* genes in *Arabidopsis thaliana*, rice and Chinese cabbage

Latin Name	Number
Arabidopsis thaliana	21
Oryza sativa	8
Brassica rapa	40

Table S4 Motif sequences detected by MEME

Motif name	Motif sequece
Motif1	KVKSCFLYCSLFPEDYEIDKEELIEYWIGEGFIDE
Motif2	KFVLLLDDIWEKVBLKKIGVPYPTRENGSKVVFTTRSREVC
Motif3	GJYGMGGVGKTTLLTQINNKF
Motif4	PLALNVIGETMASKRTVQEWRHAVDVLTSSA
motif5	TVKMHDVVREMALWIASDLGK
Motif6	LVSLRYLBLSGTKISRLPVGLQKLKKLIHLNL
Motif7	IWVVVSQDLTVEKIQDSIARKLG
Motif8	SPCFSNLSSVRISDCNGLKDLTWLLFAPN
Motif9	PFRKLEYLRLSDLPELKSIYWSPLPFPR
Motif10	IEVKCLDTDEAWELFKKKVGE

Motif1, Motif3, Motif4, Motif7, Motif10 belongs to NB-ARC; and Motif6 encodes a LRR domain.

Table S5 The promoters Cis-elements of *CC-NB-LRR* genes in Chinese cabbage.

Gene ID	Pomoter lengh	Cis-elements and Number	Function

Bra011432	1500	BIHD1OS(3)<br>GT1CONSENSUS(24);<br>GT1GMSCAM4(7)<br>SEBFCONSSTPR10A(1)<br>WRKY71OS(5)	1. **BIHD1OS:**Binding site of OsBIHD1 in disease resistance responses.<br>2. **GT1CONSENSUS:** Binding of GT-1-like factors to the PR-1a promoter influences the level of SA-inducible gene expression.<br>3. **GT1GMSCAM4:** Pathogen-and NaCl-induced expression of the SCaM-4 promoter is mediated in part by a GT-1 box that interacts with a GT-1-like transcription factor.<br>4. **SEBFCONSSTPR10A**: Binding site of the potato silencing element binding factor (SEBF) gene found in promoter of pathogenesis-related gene(PR-10a).<br>5. **WRKY71OS:** Parsley WRKY proteins bind specifically to TGAC-containing W box elements within the Pathogenesis-Related Class10 (PR-10) genes.<br>6. **WBOXATNPR1:** They were recognized specifically by salicylic acid (SA)-induced WRKY DNA binding proteins.<br>7. **WBBOXPCWRKY1**：W box"; WRKY proteins bind specifically to the DNA sequence motif.
Bra013947	265	GT1CONSENSUS(4);<br>GT1GMSCAM4(2)<br>WBOXATNPR1(2)<br>WRKY71OS(3)	
Bra026368	1500	BIHD1OS(3)<br>GT1CONSENSUS(15)<br>GT1GMSCAM4(4)<br>SEBFCONSSTPR10A(1)<br>WBOXATNPR1(3)<br>WRKY71OS(6)	
Bra031482	909	BIHD1OS(1)<br>GT1CONSENSUS(11);<br>GT1GMSCAM4(2)<br>WRKY71OS(1)	
Bra035424	1500	GT1CONSENSUS(11);<br>GT1GMSCAM4(2)<br>WRKY71OS(2)	
Bra029405	1500	BIHD1OS(3)<br>GT1CONSENSUS(7);<br>GT1GMSCAM4(4)<br>SEBFCONSSTPR10A(1)	

		WBBOXPCWRKY1(1)<br>WBOXATNPR1(2)<br>WRKY71OS(9)
Bra013134	1500	BIHD1OS(2)<br>GT1CONSENSUS(11);<br>GT1GMSCAM4(3)<br>WBBOXPCWRKY1(1)<br>WBOXATNPR1(5)<br>WRKY71OS(9)
Bra013213	1500	BIHD1OS(2)<br>GT1CONSENSUS(16);<br>GT1GMSCAM4(3)<br>WBOXATNPR1(2)<br>WRKY71OS(4)
Bra019063	1489	BIHD1OS(4)<br>GT1CONSENSUS(7);<br>GT1GMSCAM4(2)<br>SEBFCONSSTPR10A(3)<br>WBOXATNPR1(3)<br>WRKY71OS(9)
Bra036995	1500	BIHD1OS(1)<br>GT1CONSENSUS(21);<br>GT1GMSCAM4(3)<br>SEBFCONSSTPR10A(1)<br>WBBOXPCWRKY1(1)<br>WBOXATNPR1(3)

8. **MYB1LEPR**：Pti4(ERF) regulates defence-related gene expression via GCC box and non-GCC box cis elements (Myb1：GTTAGTT, G box：CACGTG)

9. **ELRECOREPCRP1**： consensus sequence of elements W1 and W2 of parsley PR1-1 and PR1-2 promoters,and WRKY1 protein binding site.

10. **TL1ATSAR**："TL1", a consensus sequence over represented in the promoter regions of all 13 NPR1-responsive ER-resident genes surveyed.

11. **Core of GCC-box** :many pathogen-responsive genes such as PDF1.2, Thi2.1, and PR4.

12. **AGCBOXNPGLB**： Conserved in most PR-protein genes

		WRKY71OS(3)
Bra018245	1500	BIHD1OS(3)<br>ELRECOREPCRP1(1)<br>GT1CONSENSUS(12);<br>GT1GMSCAM4(3)<br>MYB1LEPR(2)<br>WBBOXPCWRKY1(1)<br>WBOXATNPR1(6)<br>WRKY71OS(8)
Bra027332	1400	BIHD1OS(1)<br>ELRECOREPCRP1(1)<br>GT1CONSENSUS(10);<br>GT1GMSCAM4(3)<br>WRKY71OS(5)
Bra009882	1500	BIHD1OS(2)<br>GT1CONSENSUS(17);<br>GT1GMSCAM4(2)<br>WBOXATNPR1(2)<br>WRKY71OS(7)
Bra018834	1500	BIHD1OS(2)<br>GT1CONSENSUS(11);<br>GT1GMSCAM4(2)<br>WBOXATNPR1(3)<br>WRKY71OS(6)
Bra018835	700	BIHD1OS(3)<br>GT1CONSENSUS(12);

		GT1GMSCAM4(3)<br>WBOXATNPR1(2)<br>WRKY71OS(4)
Bra018863	1500	BIHD1OS(1)<br>GT1CONSENSUS(9);<br>GT1GMSCAM4(2)<br>SEBFCONSSTPR10A(3)<br>WBBOXPCWRKY1(1)<br>WBOXATNPR1(1)<br>WRKY71OS(5)
Bra019752	1500	BIHD1OS(2)<br>GT1CONSENSUS(11);<br>GT1GMSCAM4(1)<br>WBBOXPCWRKY1(1)<br>WBOXATNPR1(2)<br>WRKY71OS(3)
Bra019754	795	BIHD1OS(4)<br>ELRECOREPCRP1(1)<br>GT1CONSENSUS(7);<br>GT1GMSCAM4(1)<br>TL1ATSAR(1)<br>WBOXATNPR1(3)<br>WRKY71OS(6)
Bra019755	1500	BIHD1OS(2)<br>GT1CONSENSUS(11);<br>GT1GMSCAM4(3)

		SEBFCONSSTPR10A(3)<br>WBOXATNPR1(1)<br>WRKY71OS(4)
Bra026094	340	GT1CONSENSUS(5);<br>GT1GMSCAM4(3)
Bra016781	1500	BIHD1OS(3)<br>GT1CONSENSUS(13);<br>GT1GMSCAM4(2)<br>SEBFCONSSTPR10A(2)<br>WBOXATNPR1(5)<br>WBBOXPCWRKY1(2)<br>WRKY71OS(8)
Bra016782	1500	BIHD1OS(2)<br>GT1CONSENSUS(13);<br>GT1GMSCAM4(3)<br>SEBFCONSSTPR10A(1)<br>WBOXATNPR1(2)<br>WRKY71OS(8)
Bra016785	1500	BIHD1OS(1)<br>GT1CONSENSUS(8);<br>GT1GMSCAM4(1)<br>SEBFCONSSTPR10A(1)<br>WBBOXPCWRKY1(3)<br>WBOXATNPR1(12)<br>WRKY71OS(6)
Bra030778	800	BIHD1OS(2)

		ELRECOREPCRP1(1)<br>GT1CONSENSUS(8);<br>GT1GMSCAM4(1)<br>WBOXATNPR1(4)<br>WBBOXPCWRKY1(1)<br>WRKY71OS(5)
Bra030779	1500	BIHD1OS(5)<br>GT1CONSENSUS(12);<br>GT1GMSCAM4(6)<br>MYB1LEPR(1)<br>SEBFCONSSTPR10A(2)<br>WRKY71OS(7)<br>WBOXATNPR1(2)
Bra034631	1249	BIHD1OS(1)<br>ELRECOREPCRP1(1)<br>GT1CONSENSUS(19);<br>GT1GMSCAM4(8)<br>MYB1LEPR(1)<br>SEBFCONSSTPR10A(1)<br>WBOXATNPR1(3)<br>WRKY71OS(5)<br>WBBOXPCWRKY1(1)
Bra017572	526	BIHD1OS(1)<br>GT1CONSENSUS(2);<br>GT1GMSCAM4(1)<br>WRKY71OS(1)

Bra026682	1500	ELRECOREPCRP1(1)<br>GCCCORE(1)<br>GT1CONSENSUS(8);<br>GT1GMSCAM4(1)<br>WBOXATNPR1(4)<br>WRKY71OS(7)
Bra026923	1303	GT1CONSENSUS(6);<br>GT1GMSCAM4(1)<br>SEBFCONSSTPR10A(1)<br>WBBOXPCWRKY1(1)<br>WBOXATNPR1(1)<br>WRKY71OS(2)
Bra026924	644	GT1CONSENSUS(3);<br>GT1GMSCAM4(1)<br>MYB1LEPR(1)<br>WRKY71OS(1)
Bra026977	1306	BIHD1OS(4)<br>GT1CONSENSUS(11);<br>GT1GMSCAM4(2)<br>SEBFCONSSTPR10A(1)<br>WBOXATNPR1(6)<br>WRKY71OS(14)
Bra026978	1215	BIHD1OS(1)<br>ELRECOREPCRP1(1)<br>GT1CONSENSUS(10);<br>GT1GMSCAM4(3)

		SEBFCONSSTPR10A(1)<br>WBBOXPCWRKY1(1)<br>WBOXATNPR1(1)<br>WRKY71OS(6)
Bra026979	1500	BIHD1OS(2)<br>GT1CONSENSUS(7);<br>GT1GMSCAM4(4)<br>SEBFCONSSTPR10A(3)<br>WBBOXPCWRKY1(1)<br>WBOXATNPR1(1)<br>WRKY71OS(9)
Bra027097	1500	BIHD1OS(1)<br>ELRECOREPCRP1(2)<br>GT1CONSENSUS(8);<br>GT1GMSCAM4(3)<br>WBOXATNPR1(4)<br>WRKY71OS(5)
Bra027866	1500	BIHD1OS(2)<br>ELRECOREPCRP1(1)<br>GT1CONSENSUS(16);<br>GT1GMSCAM4(2)<br>WBOXATNPR1(2)<br>WRKY71OS(4)
Bra036845	1500	ELRECOREPCRP1(1)<br>GT1CONSENSUS(17);<br>GT1GMSCAM4(7)

		MYB1LEPR(1)<br>WBOXATNPR1(1)<br>WRKY71OS(1)
Bra037123	315	GT1CONSENSUS(3);<br>WBOXATNPR1(2)<br>WRKY71OS(3)
Bra037139	1500	AGCBOXNPGLB(1)<br>GT1CONSENSUS(1);<br>GT1GMSCAM4(1)<br>WBOXATNPR1(1)<br>WRKY71OS(7)
Bra002495	1500	BIHD1OS(3)<br>GT1CONSENSUS(9);<br>GT1GMSCAM4(4)<br>SEBFCONSSTPR10A(1)<br>WBBOXPCWRKY1(1)<br>WBOXATNPR1(3)<br>WRKY71OS(10)
Bra015597	1500	BIHD1OS(1)<br>GT1CONSENSUS(7)<br>WRKY71OS(2)

TableS6 The statistics of sensitive and resistant lines to DM and BR

No.	Inbred line A24 Whole area (Mean)	Lesion areas (Mean)	Rate (%)	Inbred line A95 Whole area (Mean)	Lesion areas (Mean)	Rate (%)	Inbred line A96 Whole area (Mean)	Lesion areas (Mean)	Rate (%)	Inbred line A167 Whole area (Mean)	Lesion areas (Mean)	Rate (%)
1-1	86	66	76.7%	139	27	19.4%	65	48	73.8%	91	18	19.8%
1-2	92	72	78.3%	89	17	19.1%	107	86	80.4%	117	20	17.1%
2-1	108	83	76.9%	132	26	19.7%	92	73	79.3%	66	13	19.7%
2-2	105	86	81.9%	140	29	20.7%	118	88	74.6%	68	11	16.2%
3-1	96	75	78.1%	118	23	19.5%	76	58	76.3%	126	21	16.7%
3-2	90	71	78.9%	106	21	19.8%	128	96	75%	123	23	18.7%

TableS7 Primers of qPCR

Gene No.	Forward primer Sequence (5'-3')	Reverse primer Sequence (5'-3')
Bra011432	TCTTTTTTCTTTTCGGTCGA	CGCAGTGAGAGTGACATGGT
Bra013947	AGGGAGGTTTATGCGTCGG	AGTTTGTACTCAGCACAACCTAAGC
Bra026368	TAAGTCCTTGAAATCCAGGCTAAG	TCTAAACCTTCCTTTTCGAGCA
Bra031482	CAACAAGGTTTGGAACAATTTTT	AAGTTGCATACCCCTGATCGT
Bra035424	TTTCCAGGAGCAGGTGATTC	GGATTCAATCCACAAATCGTTG
Bra029405	TGGGATTCAGGTGCAAGAG	AACGGTAAAAGTGCCACTGC
Bra013134	TATGAGTACACTAATTCTGTAAATT	CACTAGAGGAAATCCAGGACG
Bra013213	AGTGCTTTGATATATTGCATTTTGC	TTTACAGAATTAGTGTACTC
Bra019063	TGTGAAGACCATATAAAGTCAGCA	TGAGGCACCAACGACTTCTTA
Bra036995	AGAATGATCAAAGTCTTCATATGGAA	CTTTGCTCCAATACGACGAA
Bra018245	CAAGGATCTCAGGGACGATT	CTCTGAACAGTTGCGAGCC

Bra027332	TAGAGAGGCTCAACACTGCG	CCGCTGGGTTTGTAATCTGT
Bra009882	AAGTAATTGCGCGGCAAGTG	GCTTCCGCTCAATGGCTAAG
Bra018834	TGGCTTACTATTTTCTCACCAAGTC	ACTGCTTTGGTGAATGAACAAG
Bra018835	CGGTCTTACTAGCTGGAGGC	GAATGGACAAGAGAAAGAGGACA
Bra018863	CCGGATATTCCCACACTTG C	GGCTTTGCCAATCACATTGA
Bra019752	TGTTAAGTCATGTTTCCTCTATTGC	CCTCACCGATCCAATATTTAATTA
Bra019754	CGCCACGCAGTTAAGACTTT	GCTATACTTCAAAACTGGAAGAA
Bra019755	CATTGCGAAAAAACTAGGCCT	ACGTTGTGGATGTCAAGAGCT
Bra025017	TTGCATCAAACCAAACACTGA	CCCATCAACTGTTGCTTTAGC
Bra026094	TGGCTGCTTATTCGGTGATG	AGGTTTGCTTCCATTGTGTGAA
Bra016781	CAGATAATAGAGGCATACAGCCATT	TTTTAAGCTCTAATAAGCATGC
Bra016782	TACTGACCGAAGAGGTAGAAGGA	CCACATCATGCATTTTCACC
Bra016785	AGGTTGATTTTAAGTACCTTGAGGA	TGCTGAGCCTACGGAGATTG
Bra030778	AGGTGGTGGCTAGGAGGATTC	CGAGCGTCTTCTCTAAACCG
Bra030779	AGAGAGCCACTGAACCCACA	TTCCAACGTCCTTCTCCATC
Bra034631	CAAAAGTGTTGCGGTTCTTG	TCGACCACGGAAGTCTCAAT
Bra017572	CCGTTGGCAGTCAGCGTTAT	CAAAGCGTCGATTGCATACC
Bra026682	TGCAGTTTGTGGACGAGGAG	ATGTAGTAGTTTCAGTGTCCTCAAGC
Bra026923	GCTTTTCCGGAGACAGCAT	ACACAGCCAGAAGCTAAGCA
Bra026924	TCTTTTTTGTTTGGTCTTAAAGTGT	GCTAAACAATAATTAGAAAGGAAGGAA
Bra026977	AGGATCATTTCAGATGGAGGAG	ATGAGCGCATGAGTTCAGAG
Bra026978	TTCCCGGAGACAGTATTGATTA	TTTGTGGTTGTTGCTGTCTTAAC
Bra026979	AAGAGGTGGCTAGTCCTGCTG	GCATTGTTTCTTGACCAACG
Bra027097	TTCTGGTCAAGAAACTCCGAT	GCCTCTTCACGACTTCAGGT
Bra027866	AGATGCAAACGCAAAAAAGC	TCAGCGTCAAGAACAATCTCAT
Bra036845	TCCCGTGAAGAAGAAGGATT	CTGAGCTCGTAAGAACGCAC

Bra037123	TCAGGCTTACGGCATAGGAA	TCAACAGGTCGAGCACGTC
Bra037139	CTTCCTTGTCTACCTTCTATCGTTT	TCAGATTATAAGATTTTAGGAGACCTCT
Bra002495	GGTGTGTGCAGGTGAAACCA	GAGGGAACCGAAAAAATGATATAA
Bra015597	TGGTTGAACTTTAATATACGGCTG	GCTGAGCCCAAAGTGAGTGA

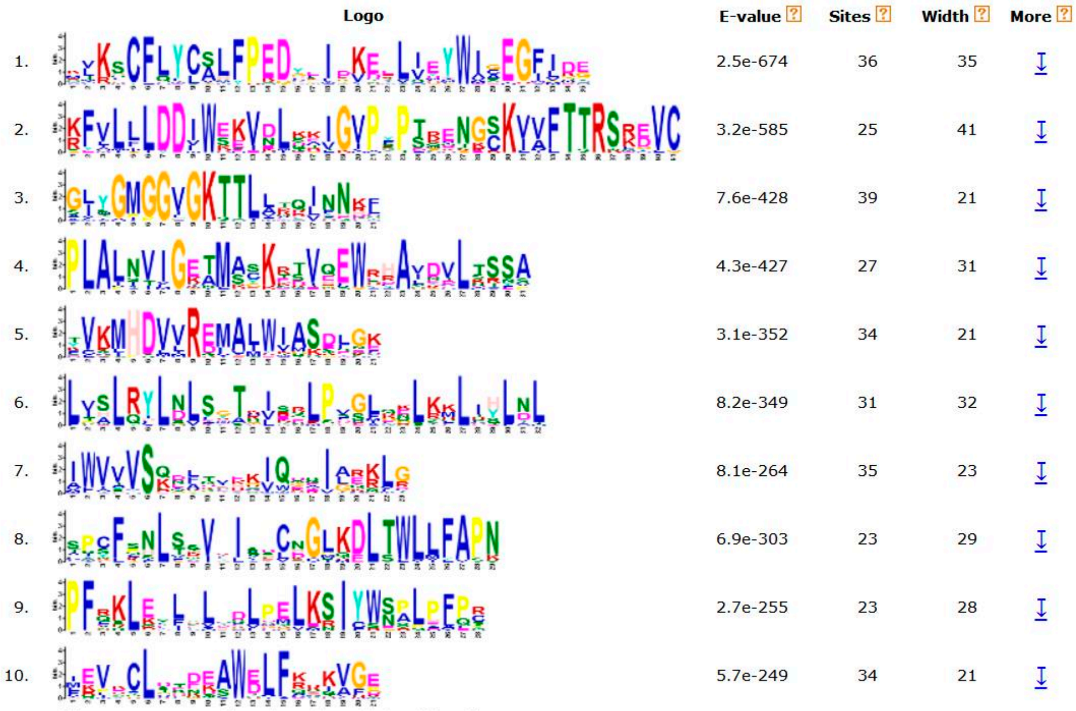

Figure S1 Sequence logos of motifs in BrCC-NBS-LRR

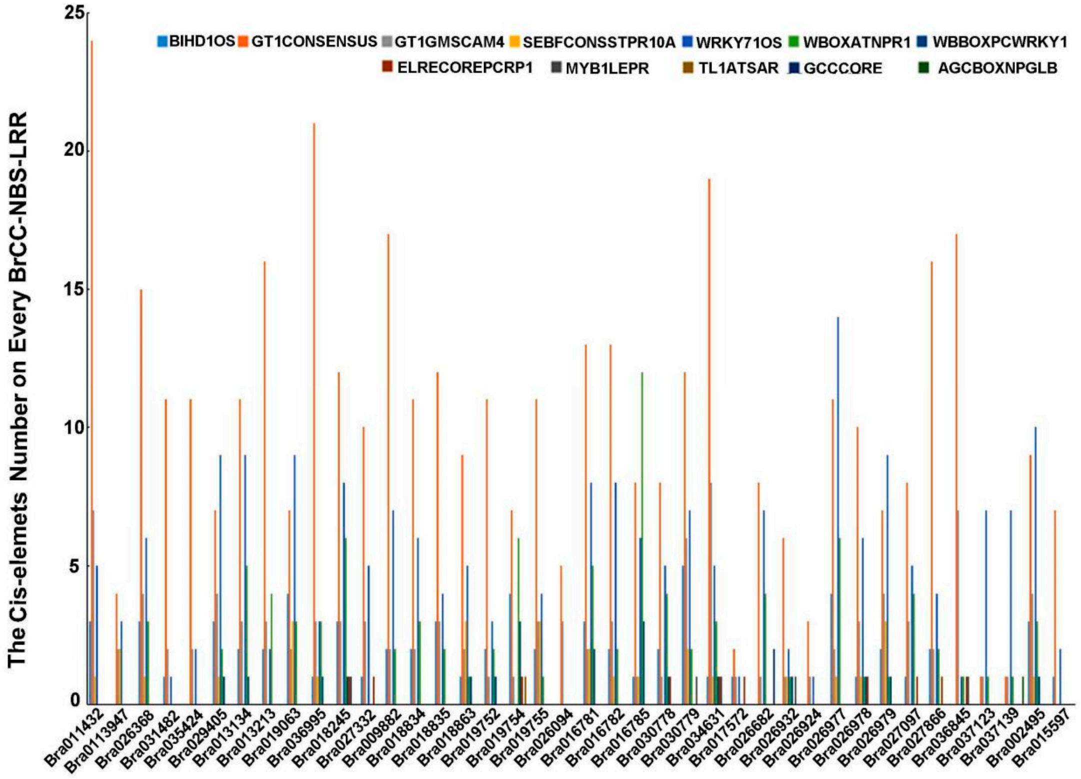

Figure S2 The number of different type cis-elements on each *BrCC-NBS-LRR* promoter.

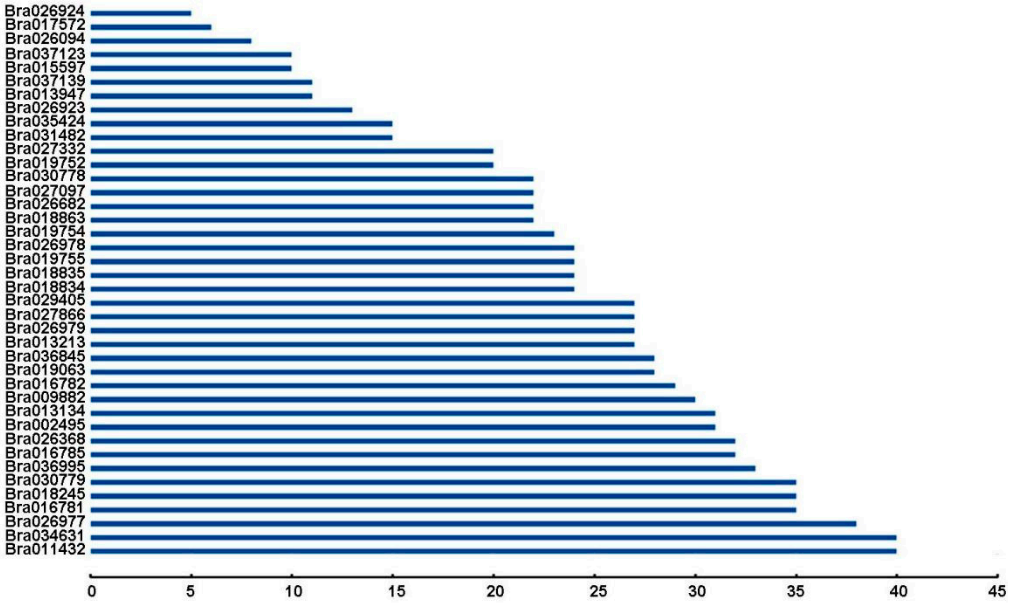

Figure S3, The total disease resistant related cis-element number on each Chinese cabbage CC-NB-LRR promoter.

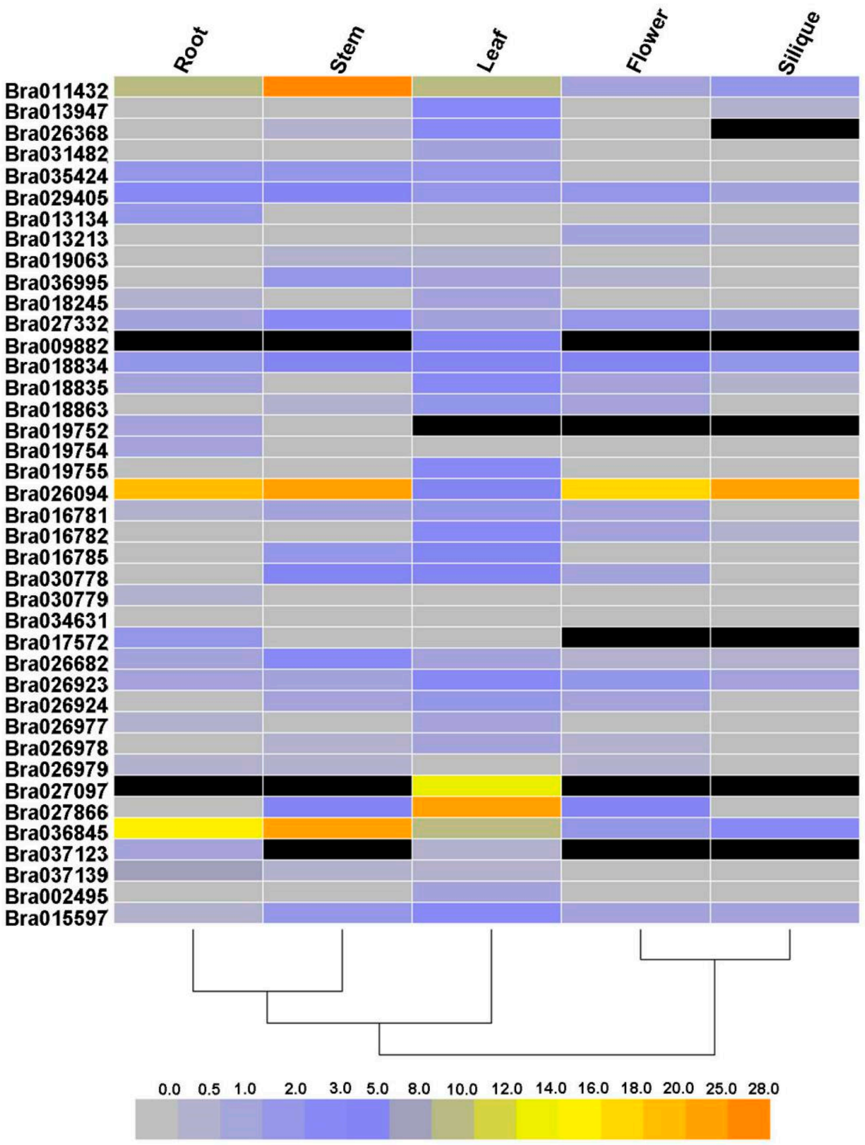

Figure S4 Hot map of *BrCC-NBS-LRRs* in root, stem, leaf, flower and silique(Black represented undected)
